# Epidemiological analysis of hospitalizations for Schizophrenia, Schizotypal Disorders and Delirium in Rio Grande do Sul over the last 5 years

**DOI:** 10.1192/j.eurpsy.2024.803

**Published:** 2024-08-27

**Authors:** L. Bardini, A. Roloff Krüger, G. Moreno Xavier, G. Fiorio Grando, J. Michelon, L. F. Alves Nascimento, J. Adames, A. T. Konzen, G. Pereira Bernd, C. Fontes Augusto, H. Wolmeister, I. Fachinetto Thoen, Y. de França, P. H. Filipin Von Muhlen, F. J. Carvalho da Costa, V. Kayser, P. H. Paesi Dutra, R. Rahal de Albuquerque, T. Garcia Furtado, G. Macelaro, A. C. Castelo, H. Vieira Rodrigues, E. Rockenbach Fidélis, D. Crusius, E. Guidugli, M. F. Valentim de Paula, Y. Marques Loureiro, E. Paiva Borsa, L. de Paula e Souza, G. Ferreira Cruz

**Affiliations:** ^1^Pontifical Catholic University of Rio Grande do Sul, Porto Alegre; ^2^Lutheran University of Brazil, Canoas; ^3^ Federal University of Health Sciences of Porto Alegre; ^4^Federal University of Rio Grande do Sul, Porto Alegre, Brazil

## Abstract

**Introduction:**

In recent years, mental health has gained prominence in public health, prompting thorough investigations into psychiatric condition trends. This study conducts a comprehensive epidemiological analysis of hospitalizations for Schizophrenia, Schizotypal, and Delirium Disorders in Rio Grande do Sul (RS) over the past five years. By revealing these patterns, it enhances our understanding of regional mental health dynamics and offers insights for intervention strategies, resource planning, and improved mental healthcare. The ultimate goal is to advance more effective and accessible mental healthcare in RS and beyond.

**Objectives:**

This study aims to analyze the prevalence and epidemiological profile of hospitalizations due to psychiatric disorders to assist in the diagnosis and outcome of affected patients.

**Methods:**

A cross-sectional, descriptive, retrospective, and quantitative study was conducted regarding hospitalizations for Schizophrenia, Schizotypal Disorders, and Delirium in the state of RS between January 2018 and November 2022. Data were collected from the Department of Informatics of the Brazilian Unified Health System (DATASUS) in the “Hospital Information System of SUS” section, focusing on the nature of care, age group, gender, and ethnicity of the patients. The information was aggregated over the five-year period based on the four mentioned descriptors and subsequently analyzed to establish a profile of hospitalizations during that period.

**Results:**

The analysis spans from 2018 to 2022, encompassing a total of 28,345 hospitalizations. In 2019, there was the highest number of cases (22.21%), followed by 2018 (21.08%). Urgent care admissions constituted 85.34% of the total. The age group most affected was 35 to 39 years (11.8%). Men were more affected than women (60.18%), and the majority of hospitalizations were among the Caucasian ethnicity (75.12%). The average length of stay was 23.7 days, and the mortality rate stood at 0.26%.

**Conclusions:**

The increasing trend in hospitalizations, peaking in 2019, highlights the need for preventive measures. Urgent admissions (85.34%) underscore the demand for accessible mental health resources. Men in the 35 to 39 age group are disproportionately affected, suggesting specific risk factors. The predominance of Caucasian ethnicity emphasizes the need for culturally sensitive care. A longer average length of stay (23.7 days) underscores treatment complexity, while a low mortality rate (0.26%) signals effective medical care. In essence, these findings inform tailored mental health policies to enhance service quality and prioritize patient-centered approaches.

**Disclosure of Interest:**

None Declared

